# The Impact of the Thoracolumbar Junction Distraction Technique on Reducing Proximal Junctional Kyphosis: A Comparative Pre- and Post-Implementation Study in Adult Spinal Deformity Surgery

**DOI:** 10.3390/medicina61071192

**Published:** 2025-06-30

**Authors:** Dae-Woong Ham, Hyun Suk Shin, Ohsang Kwon, Sang-Min Park, Ho-Joong Kim

**Affiliations:** 1 Department of Orthopaedic Surgery, Chung-Ang University Hospital, College of Medicine, Chung-Ang University, Seoul 06973, Republic of Korea; hamdgogo@gmail.com; 2 Spine Center and Department of Orthopaedic Surgery, Seoul National University College of Medicine, Seoul National University Bundang Hospital, Seongnam 13605, Republic of Korea; carlshin@naver.com (H.S.S.); ormssang@gmail.com (O.K.); grotyx@gmail.com (S.-M.P.)

**Keywords:** kyphosis, thoracolumbar junction distraction technique, spinal curvatures/surgery

## Abstract

*Background and Objectives*: Proximal junctional kyphosis (PJK) remains a significant complication in adult spinal deformity (ASD) surgery, often resulting in severe clinical consequences. This study evaluates the effectiveness of the thoracolumbar junction (TLJ) distraction technique in reducing PJK incidence, with a focus on its potential to preserve sagittal alignment and mitigate mechanical stress at the proximal junction. *Materials and Methods*: This retrospective cohort study included 122 patients who underwent ASD surgery between February 2018 and June 2022. Patients were stratified into a control group and an intervention group based on the application of the TLJ distraction technique. Radiographic and clinical outcomes, including proximal junctional angle (PJA), thoracolumbar angle (TLA), and PJK incidence, were assessed one year postoperatively. Statistical analyses were performed using *t*-tests and chi-square tests. *Results*: The incidence of PJK was significantly lower in the intervention group compared with that in the control group (24.6% vs. 44.3%, *p* = 0.036). Additionally, the intervention group exhibited a significant reduction in postoperative TLA (−10.6° ± 6.3° vs. −6.8° ± 6.8°, *p* = 0.002) and ΔTLA (2.6° ± 9.0° vs. −2.4° ± 9.5°, *p* = 0.003). Although improvements in radiographic parameters were associated with a trend toward reduced rates of proximal junctional failure (PJF), statistical significance was not achieved. *Conclusions*: The TLJ distraction technique effectively reduces PJK incidence by optimizing thoracolumbar alignment and minimizing abrupt sagittal transitions. This simple and reproducible approach presents a promising strategy for mitigating proximal junctional complications in ASD surgery, warranting further validation in multicenter trials.

## 1. Introduction

Adult spinal deformity (ASD) is a complex pathological condition that profoundly affects patient quality of life [1,2,3]. In recent years, surgical intervention has emerged as a principal therapeutic approach, demonstrating significant clinical efficacy [4,5,6]. A variety of surgical techniques for deformity correction have been employed, each presenting distinct biomechanical advantages and limitations [7,8,9]. However, the high incidence of postoperative complications, including proximal junctional kyphosis (PJK) and proximal junctional failure (PJF), remains a critical concern [10,11]. These complications may manifest as progressive kyphotic deformity, mechanical instability, and, in severe cases, neurological impairment, including paralysis [12].

To mitigate the risk of PJK, multiple prophylactic strategies have been proposed, including cement augmentation at the uppermost instrumented vertebra (UIV), reinforced instrumentation for UIV fixation, and the augmentation of ligamentous structures in the vicinity of the UIV [9,13,14]. Despite these efforts, a comprehensive understanding of the biomechanical factors contributing to PJK development, particularly in relation to the thoracolumbar junction (TLJ), remains incomplete.

The TLJ represents a biomechanically critical transitional region, rendering it particularly susceptible to stress accumulation and subsequent mechanical failure following spinal deformity correction. Excessive correction at the TLJ can result in stress concentration at the proximal terminus of the fusion construct, thereby increasing the likelihood of ligamentous failure and associated mechanical complications. However, limited research has been conducted to elucidate the specific role of TLJ biomechanics in the prevention of PJK and related complications [15].

Emerging retrospective analyses have identified a correlation between PJK development and alterations in thoracolumbar alignment parameters, including the thoracolumbar angle (TLA) and thoracolumbar slope (TLS) [16]. Notably, changes in TLS may induce compensatory thoracic kyphosis, thereby exacerbating the risk of PJK [17]. In response to these findings, we developed and implemented the TLJ distraction technique, a novel approach designed to attenuate abrupt alterations in TLA and minimize mechanical stress at the proximal fusion segment.

The present study employs a retrospective cohort design with a pre- and post-implementation framework to assess the efficacy of the TLJ distraction technique in reducing PJK incidence. By systematically comparing clinical and radiographic outcomes before and after the introduction of this technique in ASD surgery, we aimed to provide valuable insights into its biomechanical advantages and potential clinical applications.

## 2. Materials and Methods

### 2.1. Study Design

This study was a retrospective observational cohort study conducted at a tertiary hospital between February 2018 and June 2022. All procedures were conducted at Seoul National University Bundang Hospital (SNUBH), a tertiary academic medical center. The study employed a comparative pre- and post-implementation design to evaluate the TLJ distraction technique, which was introduced in November 2020. Patients who underwent surgery prior to the implementation of this technique constituted the control group, for which data were analyzed retrospectively. Those who underwent surgery thereafter were categorized as the intervention group and were prospectively followed. The primary outcome measure was the incidence of PJK within one year postoperatively.

**Figure 1:**
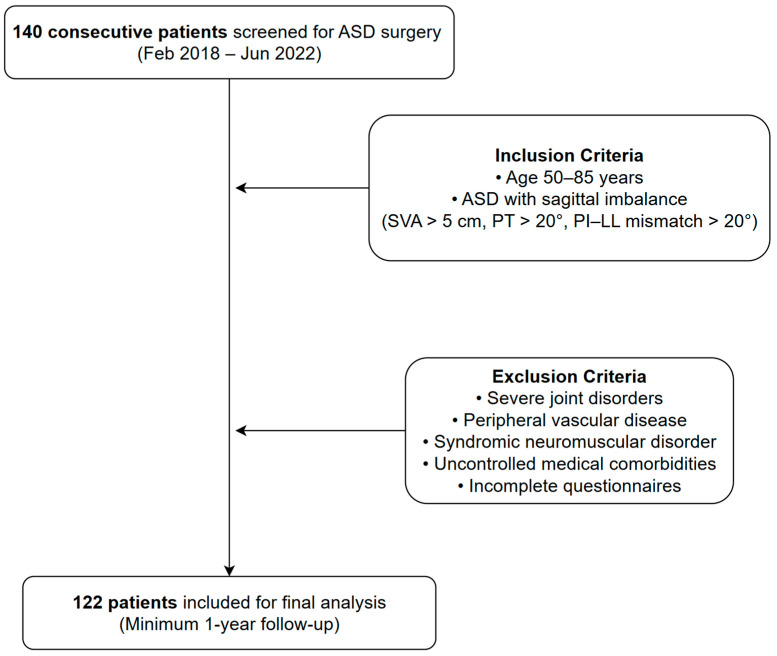
Flowchart of patient enrollment and application of inclusion/exclusion criteria.

The inclusion criteria were as follows: (1) adult patients aged 50 to 85 years and (2) individuals scheduled for corrective surgery for ASD with sagittal imbalance. ASD was defined by the presence of at least one of the following radiographic parameters on lateral standing radiographs: sagittal vertical axis (SVA) >5 cm, pelvic tilt (PT) >20°, or pelvic incidence (PI) lumbar lordosis (LL) mismatch >20°. Patients were excluded if they met any of the following criteria: (1) severe joint disorders impairing ambulation, (2) peripheral vascular disease, (3) any syndromic neuromuscular disorder, (4) uncontrolled medical comorbidities, including sepsis or malignancy, or (5) inability to complete patient-reported outcome questionnaires. A total of 140 consecutive patients were screened based on these criteria. Following the exclusion of 18 patients, the final analysis included 122 patients, all of whom had a minimum follow-up period of one year (Figure 1).

### 2.2. Surgical Procedures and TLJ Distraction Technique

The correction target was to achieve a PI–LL mismatch within 10°. To attain the target lumbar lordosis correction, multilevel posterior column osteotomies (PCOs) or pedicle subtraction osteotomy (PSO) was performed as indicated. Patients were positioned on a Jackson table to optimize lumbar lordosis prior to correction. The selection of fusion levels was meticulously determined based on both coronal and sagittal deformities. All surgical procedures involved long-level fusions (>5 levels), with the UIV positioned in the lower thoracic region and the lower instrumented vertebra (LIV) extending to S1 or the pelvis. To prevent placement of the UIV at the apex of the thoracic kyphotic curve, the UIV level was chosen at either T9 or T10. Interbody fusion with a cage and bone graft was performed in the lower lumbar regions, and a cantilevering rod technique was employed to facilitate deformity correction. In all cases, cobalt-chrome rods were used as primary rod constructs. In instances requiring multi-rod constructs (three- or four-rod configurations), titanium rods were utilized as supplemental constructs. All surgeries were performed by a single experienced spine surgeon (the leading author) to ensure procedural consistency and eliminate inter-operator variability.

**Figure 2:**
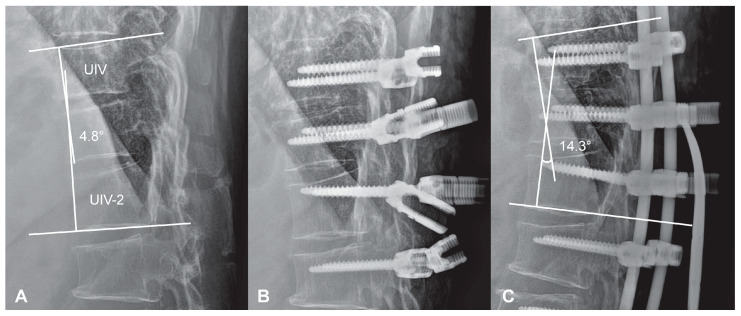
Intraoperative application of the TLJ distraction technique. (**A**) Preoperative prone lateral radiograph showing the baseline kyphotic angle between UIV and UIV–2 (4.8°). (**B**) Pedicle screws inserted at UIV, UIV–1, and UIV–2 with sagittal orientation tailored to accommodate a pre-contoured rod replicating mild kyphosis. Screw heads are aligned to facilitate gradual distraction without anterior over-angulation. (**C**) Final construct demonstrating controlled kyphotic correction (14.3°) achieved via distraction between UIV–UIV–1 and UIV–1–UIV–2 under fluoroscopic guidance and sequential tightening.

The TLJ distraction technique was implemented in a systematic manner to minimize abrupt changes in thoracolumbar alignment and mitigate mechanical stress at the proximal segment of the fusion construct. The following stepwise protocol was employed to ensure consistency and reproducibility (Figure 2):

- Preoperative planning: The TLA and TLS were measured on standing lateral radiographs to define each patient’s baseline physiological curvature at the thoracolumbar junction. These values served as intraoperative references for targeted alignment.
- Positioning: Patients were placed prone on a Jackson table with adequate lumbar support to prevent excessive flattening. Lateral fluoroscopy or intraoperative plain radiograph was utilized to ensure the preservation of the native TLA during positioning.
- Screw placement: Pedicle screws were inserted at the UIV, UIV-1, and UIV-2 levels with close attention to sagittal orientation. Screw heads were aligned to accommodate a pre-contoured rod that replicated a mild kyphotic curve, avoiding aggressive anterior angulation.
- Rod contouring and placement: A pre-bent cobalt-chrome rod, shaped to match the intended kyphotic curvature of the TLJ, was positioned across the UIV to UIV-2 levels.
- Controlled distraction: Distraction was sequentially applied between UIV–UIV-1 and UIV-1–UIV-2 using a rod distractor or compressor system. Under radiographic guidance, distraction was adjusted with the goal of producing a segmental kyphosis 5° to 10° greater than the angle measured between UIV and UIV-2 in the prone position. Set screws were progressively tightened during this process to maintain the desired curvature and ensure gradual stress distribution.
- Final locking: After confirming smooth sagittal alignment without abrupt transitions or focal angulation, all screws were securely locked.

This standardized approach was designed to preserve the natural curvature of the thoracolumbar junction, minimize proximal construct rigidity, and reduce the risk of junctional complications.

### 2.3. Clinical and Radiographic Outcome Measurement

Clinical outcomes were assessed using the Oswestry Disability Index (ODI) and the EuroQol-5 Dimension (EQ-5D) score. The ODI is a patient-reported outcome measure designed to evaluate back-specific functional impairment based on a 10-item questionnaire, with total scores converted into a percentage scale. The EQ-5D is a standardized instrument for measuring health-related quality of life across five domains: mobility, self-care, usual activities, pain/discomfort, and anxiety/depression. EQ-5D scores range from 0 to 1.00, where 1.00 denotes “full health” and 0 represents the “worst health.”

**Figure 3:**
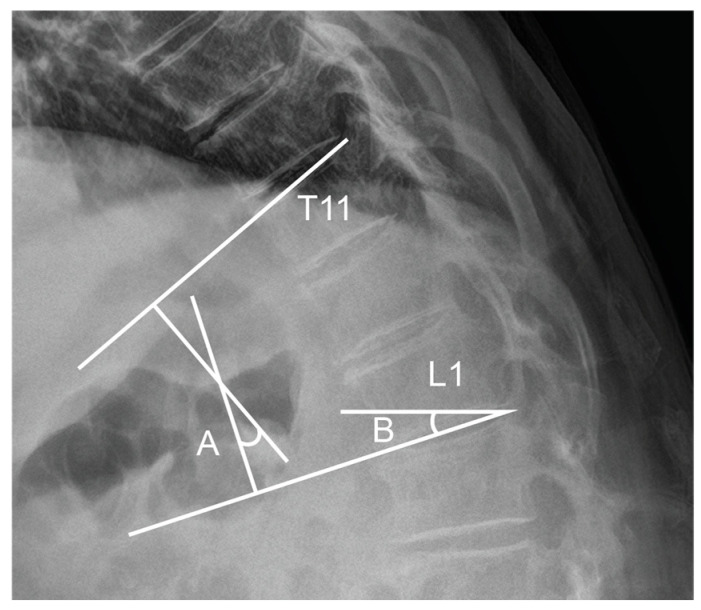
Measurement of TLA and TLS. TLA is measured as the Cobb angle between the superior endplate of T11 and the inferior endplate of L1 (A), while TLS is defined as the angle between the superior endplate of L1 and a horizontal reference line (B).

Radiologic parameters, including SVA, LL, PT, sacral slope (SS), PI, TLA, and TLS, were measured in the standing position preoperatively and at two weeks postoperatively using biplanar radiographic imaging (EOS imaging, Paris, France). The TLA was determined as the Cobb angle between the superior endplate of T11 and the inferior endplate of L1, while the TLS was defined as the angle between the superior endplate of L1 and a horizontal reference line (Figure 3). Bone mineral density (BMD) was assessed using dual-energy X-ray absorptiometry (DEXA, Horizon DXA system, Hologic, Inc., Marlborough, MA, USA), with the lowest recorded value among the lumbar vertebrae and hip joints, excluding Ward’s triangle. PJK was defined as a postoperative proximal junctional angle exceeding 10° with an increase of more than 10° compared to the preoperative measurement. PJF was defined as the presence of PJK in conjunction with one or more of the following: fracture at the UIV or UIV + 1, neurological deterioration, or the need for revision surgery. To mitigate bias associated with variable follow-up durations, PJK and PJF incidences were evaluated based on radiographs obtained at a one-year postoperative follow-up.

### 2.4. Statistical Analysis

Data are presented as means ± standard deviations, medians with interquartile ranges [Q1–Q3], or absolute numbers with corresponding percentages. The normality of continuous variables was assessed using the Shapiro–Wilk test. Depending on the distribution, between-group comparisons were performed using either the independent *t*-test or the Mann–Whitney *U* test. Mean comparisons for continuous variables were conducted using an independent *t*-test, while categorical variables were analyzed using the chi-square test.

A multivariable logistic regression analysis was conducted to adjust for potential confounding variables, including age, sex, BMI, BMD, hand grip strength (HGS), spinopelvic parameters (SS, PT, PI–LL mismatch), UIV levels, and the presence of a PSO. The dependent variable was the incidence of PJK. Odds ratios (ORs) and 95% confidence intervals (CIs) were calculated by exponentiating the regression coefficients. A *p*-value < 0.05 was considered statistically significant. All statistical analyses were performed using R version 4.4.2 (R Development Core Team, Vienna, Austria), with statistical significance set at *p* < 0.05.

## 3. Results

### 3.1. Demographic and Baseline Characteristics

A total of 122 patients were included in the study, with 61 patients allocated to the control group and 61 to the intervention group. No significant differences were observed between the two groups in terms of demographic and baseline clinical characteristics, including sex, age, BMI, BMD, HGS, and spinopelvic parameters (Table 1). Additionally, surgical factors, such as the selection of the UIV level (T9 vs. T10) and osteotomy type (PCO vs. PSO), were comparable between the groups.

### 3.2. Proximal Junctional Kyphosis: Failure and Clinical Outcomes

The overall incidence of PJK was 34.4% (42/122). Following the implementation of the TLJ distraction technique, the incidence of PJK significantly decreased from 44.3% in the control group to 24.6% in the intervention group (*p* = 0.036). Similarly, the incidence of PJF demonstrated a downward trend, declining from 28.6% to 17.3%; however, this difference did not reach statistical significance (*p* = 0.146) (Table 1).

Both groups demonstrated improvement in ODI and EQ-5D scores from baseline to 12 months postoperatively, but there were no statistically significant between-group differences (ODI: 17.0 [8.0–22.0] vs. 16.0 [9.0–24.0], *p* = 0.870; EQ-5D: 0.410 [0.093–0.553] vs. 0.410 [0.081–0.568], *p* = 0.881, Table 1).

### 3.3. Spinopelvic and Thoracolumbar Junction Parameters

There were no significant differences between the two groups in preoperative or immediate postoperative spinopelvic parameters, including SS, PI, LL, and SVA. However, postoperative TLA was significantly lower in the intervention group compared to the control group (−10.6° ± 6.3° vs. −6.8° ± 6.8°, *p* = 0.002) (Table 2). Additionally, the change in TLA (ΔTLA) differed significantly between groups, with the control group exhibiting a flatter or more lordotic curvature (2.1° [−2.7–8.7°] vs. −3.8° [−9.4–1.7°], *p* = 0.002).

### 3.4. Multivariable Analysis for Risk Factors of PJK

To identify independent predictors of PJK, a multivariable logistic regression analysis was conducted. The following variables were included in the model: age, sex, BMI, BMD, HGS, preoperative spinopelvic parameters (SS, PT, PI–LL mismatch), postoperative PI–LL mismatch, UIV level, and the presence of a PSO. The application of the TLJ distraction technique (group variable) was also included as a key covariate.

The regression analysis revealed that the TLJ distraction technique was independently associated with a reduced risk of PJK (OR = 0.39, 95% CI: 0.15–0.97, *p* = 0.044). In contrast, increasing age was significantly associated with a higher risk of PJK (OR = 1.10, 95% CI: 1.02–1.18, *p* = 0.017). Other variables, including BMD, BMI, HGS, spinopelvic parameters, and surgical factors, did not show statistically significant associations with PJK incidence.

These results suggest that the observed reduction in PJK following the implementation of the TLJ distraction technique is not solely attributable to differences in baseline characteristics or surgical indications, but reflects the independent effect of the technique itself. A summary of the regression model is presented in Table 3.

## 4. Discussion

This study demonstrates that the TLJ distraction technique significantly reduces the incidence of PJK in patients undergoing ASD surgery. Specifically, the incidence of PJK was reduced from 44.3% in the control group to 24.6% in the intervention group (*p* = 0.036), underscoring the clinical efficacy of this technique. Furthermore, patients in the intervention group exhibited significantly lower postoperative TLA and ΔTLA compared to those in the control cohort, suggesting an improved biomechanical profile. Although the incidence of PJF did not reach statistical significance, the favorable trend suggests that further research with larger cohorts or longer follow-ups could clarify the technique’s impact on more severe junctional pathologies.

In order to strengthen the validity of our findings and address potential confounding factors inherent to the retrospective design, we conducted a multivariable logistic regression analysis adjusting for demographic, radiological, and surgical variables. Notably, the TLJ distraction technique remained independently associated with a reduced risk of PJK (OR = 0.39, 95% CI: 0.15–0.97, *p* = 0.044) even after adjustment, reinforcing its efficacy beyond baseline group differences. Age was also identified as an independent predictor of PJK, consistent with prior reports linking older age with decreased compensatory capacity and ligamentous resilience [10]. Other factors, including spinopelvic alignment and surgical variables such as PSO, did not show significant associations. These results provide further support that the reduction in PJK incidence was not merely due to selection bias or uncontrolled confounders, but reflects a true preventative effect of the TLJ distraction technique.

The TLJ represents a biomechanically vulnerable transition zone between the rigid thoracic spine and the more flexible lumbar spine [18,19]. Excessive flattening or lordotic alignment at this region predisposes patients to junctional complications due to stress concentration and ligamentous failure. The TLJ distraction technique preserves the physiological curvature of this junction, thereby mitigating excessive mechanical strain and reducing proximal construct tension. These findings are consistent with previous research identifying ΔTLA as a critical risk factor for PJK and emphasizing the biomechanical advantage of maintaining controlled TLA to prevent stress accumulation at the TLJ [15,16]. The present study further reinforces this perspective by providing additional clinical evidence supporting the application of TLJ distraction.

Previous studies have emphasized the importance of maintaining sagittal alignment to minimize postoperative complications and improve quality of life in ASD patients. Recent research has further highlighted the critical role of TLJ alignment in preventing PJK. Studies found that excessive correction in the TLJ region can increase mechanical stress at the proximal junction, predisposing patients to junctional kyphosis and long-term alignment loss [20,21,22]. Pizones et al. [23] demonstrated that patients with greater pelvic rotation reserve and balanced lumbar lordosis were better able to compensate for PJK, whereas those with excessive TLJ correction had limited compensatory ability, leading to progressive kyphotic deformity. These findings underscore the need to avoid overcorrection in the TLJ area and to ensure an optimal sagittal profile that allows for adequate biomechanical adaptation postoperatively.

Insights from studies examining normal TLJ alignment in healthy adults further support the notion that this region exhibits gradual segmental motion toward the lumbar spine, with a mean TLS of −12.8 ± 7.8° [24]. Attempts to impose an overly flat or lordotic configuration may exceed the physiological threshold, inducing compensatory limitations or elevated mechanical stress at the proximal junction. Similarly, lordosis orientation (LO) variables—such as the angle of the uppermost instrumented vertebra to the femur or the degree of PI–LL mismatch—have been correlated with higher rates of PJK in large cohort analyses [25]. Collectively, these results imply that an over- or under-corrected lumbar lordosis, coupled with excessive TLJ manipulation, may compromise the delicate balance of spinal segments and exacerbate junctional problems. One primary mechanism driving PJK is the progressive kyphotic deformity that develops in the proximal segments above the UIV through what is often referred to as “reciprocal change” [26,27,28]. Hence, maintaining the cone of economy while also avoiding excessive lordotic correction at the TLJ is crucial for preventing the proximal segments from experiencing progressive kyphotic forces arising from reciprocal change.

In light of the persistent challenge posed by PJK, multiple alternative or supplementary prophylactic methods have been investigated, including ligament augmentation [9], bone cement reinforcement at the UIV [29], and the “Soft Landing” technique employing hooks or sublaminar bands [30]. These strategies can redistribute mechanical loads and stabilize osteoporotic vertebrae but often require additional surgical steps, implants, or materials. In particular, cement augmentation techniques may be associated not only with increased complexity and cost, but also with potential complications such as cement leakage, pulmonary embolism, adjacent vertebral fracture, and infection, as reported by Moldovan et al. [31].

By contrast, the TLJ distraction technique integrates seamlessly into standard long-level fusions, relying mainly on adjusting alignment and distraction force at the transitional segments. Although technically straightforward, this technique requires a solid understanding of thoracolumbar biomechanics, careful rod contouring, and real-time fluoroscopic assessment. In our study, all procedures were performed by a single experienced spine surgeon to minimize variability related to the learning curve.

Critically, while lumbar lordosis has long been recognized as a key intraoperative parameter that surgeons can actively manipulate in ASD procedures, our findings highlight that TLJ orientation is likewise modifiable and can be verified intraoperatively via imaging. This direct control over TLJ curvature allows for proactive, targeted alignment adjustments—thereby reducing the abrupt mechanical stress shifts that precipitate PJK. Although existing approaches for PJK prevention, such as soft tissue preservation or cement augmentation, may add complexity or cost, the TLJ distraction technique remains straightforward, reproducible, and relatively cost-effective within the standard surgical workflow.

Despite its strengths, this study has several limitations. The retrospective design and single-center nature may constrain the generalizability of the findings. Additionally, the focus on lower thoracic levels as the UIV limits the applicability of these results to cases involving upper thoracic fixation. Although we demonstrated sufficient statistical power (98.7%) to detect differences in PJK incidence with a moderate to large effect size (w = 0.419), the follow-up duration of one year may not fully capture late-onset PJK or long-term complications such as PJF and revision surgery. Furthermore, the study may have been unable to detect differences in PJF rates, which did not reach statistical significance. Given that PJF occurs less frequently than PJK, a larger sample size or extended follow-up period may be required to clarify its true impact. Finally, although multivariable logistic regression was used to adjust for potential confounders, the retrospective and non-randomized design may not have eliminated residual bias. Future multicenter, randomized controlled trials with 5- to 10-year follow-ups are warranted to validate the durability of the TLJ distraction technique and confirm its long-term efficacy in PJK and PJF prevention.

As surgical paradigms continue to evolve in ASD management, future research should delve deeper into the biomechanical mechanisms underlying the TLJ distraction technique, potentially through advanced computational modeling and cadaveric analyses. Such efforts would clarify how controlled distraction specifically mitigates PJK-inducing stresses and guide refinements to operative protocols. In parallel, integrating TLJ distraction with other prophylactic measures—such as soft tissue preservation, dynamic stabilization, or multi-rod constructs—may offer synergistic benefits, particularly in osteoporotic or high-risk patients. Long-term prospective studies assessing revision rates and patient-reported outcomes are essential to capture the full clinical impact of these combined strategies. Further enhancements may stem from sophisticated intraoperative navigation and 3D imaging, which enable real-time assessment and the fine-tuning of alignment parameters (e.g., TLA, TLS). Moreover, minimally invasive surgery (MIS) approaches increasingly aim to reduce perioperative morbidity and preserve paraspinal musculature; blending MIS principles with targeted maneuvers like TLJ distraction could achieve critical alignment goals while minimizing soft tissue damage. In the same vein, adjunctive interventions—such as prophylactic vertebroplasty—could reinforce osteoporotic vertebrae, reducing the likelihood of proximal junctional failure. By exploring these convergent avenues, future investigations have the potential to delineate how best to optimize sagittal alignment, minimize mechanical complications, and improve long-term clinical outcomes for patients undergoing ASD correction.

## 5. Conclusions

The TLJ distraction technique has demonstrated efficacy in reducing the incidence of PJK in ASD surgery by optimizing thoracolumbar alignment and minimizing abrupt sagittal transitions. This approach, characterized by its simplicity and reproducibility, represents a viable strategy for mitigating proximal junctional complications while avoiding additional surgical complexity.

## Figures and Tables

**Table 1:** Demographic characteristics and clinical outcomes pre- and post-thoracolumbar junction distraction technique implementation.

TLJ Distraction (N)	Before (61)	Implemented (61)	Total (122)	*p*
Sex, Female	52 (85.2%)	58 (95.1%)	110 (90.2%)	0.128
Age	71.9 ± 7.9	71.9 ± 5.7	71.9 ± 6.9	0.990
BMI (kg/m^2^)	26.1± 3.7	26.3 ± 3.8	26.2± 3.7	0.787
BMD (g/cm^2^)	0.554 [0.495–0.609]	0.560 [0.513–0.618]	0.559 [0.506–0.615]	0.453
HGS (N)				
Preoperative	18.0 ± 6.7	17.0 ± 6.3	17.5 ± 6.5	0.408
Sacral slope (°)	16.5 ± 9.8	20.1 ± 12.9	18.3 ± 11.5	0.081
Pelvic tilt (°)	33.2 ± 10.6	30.1 ± 9.3	31.6 ± 10.1	0.089
Pelvic incidence (°)	49.7 ± 10.5	50.1 ± 10.9	49.9 ± 10.7	0.833
Lumbar lordosis (°)	−4.4 ± 19.8	0.6 ± 18.7	−1.9 ± 19.4	0.153
PI–LL	54.0 ± 20.4	49.4 ± 18.0	51.7 ± 19.3	0.188
SVA (cm)	159.0 ± 67.9	151.8 ± 78.6	155.4 ± 73.2	0.587
Immediate postoperative				
Sacral slope (°)	29.3 ± 11.4	26.4 ± 9.4	27.8 ± 10.5	0.119
Pelvic tilt (°)	22.2 ± 11.1	21.7 ± 8.0	22.0 ± 9.7	0.790
Lumbar lordosis (°)	41.4 ± 15.6	37.4 ± 8.3	39.4 ± 12.6	0.087
PI–LL	10.2 ± 14.9	10.6 ± 8.2	10.4 ± 12.0	0.843
SVA	33.8 ± 38.3	40.8 ± 36.1	37.3 ± 37.2	0.305
UIV level				0.128
T9	3 (4.9%)	9 (14.8%)	12 (9.8%)	
T10	58 (95.1%)	52 (85.2%)	110 (90.2%)	
Osteotomy				0.551
3-level PCO	41 (67.2%)	45 (73.8%)	86 (70.5%)	
PSO	20 (32.8%)	16 (26.2%)	36 (29.5%)	
PJK	27 (44.3%)	15 (24.6%)	42 (34.4%)	**0.036**
PJF	18 (29.5%)	12 (19.7%)	30 (24.6%)	0.293
Preoperative				
ODI	25.0 ± 7.4	23.5 ± 8.0	24.3 ± 7.7	0.287
EQ-5D	0.093 [0.081–0.355]	0.205 [0.081–0.410]	0.196 [0.081–0.410]	0.179
12-months postoperative				
ODI	17.0 [8.0–22.0]	16.0 [9.0–24.0]	16.0 [9.0–22.0]	0.870
EQ-5D	0.410 [0.093–0.553]	0.410 [0.081–0.568]	0.410 [0.081–0.558]	0.881

Data are presented as mean ± SD, median [IQR], or n (%), as appropriate. Values in bold are considered statistically significant (*p* < 0.05). Abbreviations: BMI, body mass index; BMD, bone mineral density; HGS, hand grip strength; SS, sacral slope; PT, pelvic tilt; PI, pelvic incidence; LL, lumbar lordosis; PI–LL, pelvic incidence minus lumbar lordosis; SVA, sagittal vertical axis; UIV, upper instrumented vertebra; PCO, posterior column osteotomy; PSO, pedicle subtraction osteotomy; PJK, proximal junctional kyphosis; PJF, proximal junctional failure; ODI, Oswestry Disability Index; EQ-5D, EuroQol-5 Dimension.

**Table 2:** Comparison of spinopelvic and thoracolumbar junction parameters before and after TLJ distraction technique implementation.

TLJ Distraction (N)	Before (61)	Implemented (61)	Total (122)	*p*
Preoperative				
PJA (°)	2.2 ± 5.2	2.2 ± 6.2	2.2 ± 5.7	0.973
TLA (°)	−6.2 [−17.2–−1.8]	−7.5 [−15.9–0.1]	−6.6 [−16.5–−1.2]	0.710
TLS (°)	20.5 ± 16.4	16.5 ± 15.8	18.5 ± 16.2	0.178
Immediate postoperative				
PJA (°)	7.3 ± 5.8	7.0 ± 6.1	7.1 ± 5.9	0.734
TLA (°)	6.3 [−9.7–−3.0]	−9.5 [−15.8–−6.6]	−7.9 [−12.8–−4.2]	**0.001**
TLS (°)	−11.3 ± 7.3	−11.7 ± 7.1	−11.5 ± 7.2	0.771
Postoperative change				
PJA (°)	5.2 ± 4.8	4.8 ± 6.4	5.0 ± 5.7	0.696
TLA (°)	2.1 [−2.7–8.7]	−3.8 [−9.4–1.7]	−0.9 [−6.4–5.6]	**0.00** **2**
TLS (°)	−31.8 ± 15.1	−28.2 ± 15.9	−30.0 ± 15.5	0.204

Data are presented as mean ± SD, median [IQR], or n (%), as appropriate. Values in bold are considered statistically significant (*p* < 0.05). Abbreviations: PJA, proximal junctional angle; TLA, thoracolumbar angle; TLS, thoracolumbar slope.

**Table 3:** Multivariable logistic regression analysis identifying independent risk factors for PJK.

Variable	Coefficient (β)	*p*-Value	Odds (95% CI)
Age	0.0923	**0.017**	1.10 (1.02–1.18)
Sex (Male)	0.4142	0.636	1.54 (0.28–8.56)
BMI	−0.0536	0.441	0.95 (0.83–1.09)
BMD	−4.791	0.095	0.01 (0.00–2.03)
HGS	0.0186	0.598	1.02 (0.95–1.09)
Group (TLJ distraction)	−0.9524	**0.044**	0.39 (0.15–0.97)
Preoperative SS	0.2798	0.438	1.35 (0.66–2.74)
Preoperative PT	0.2852	0.442	1.36 (0.65–2.85)
Preoperative PI	−0.3195	0.388	0.73 (0.35–1.50)
Preoperative PI–LL	−0.3012	0.412	0.99 (0.96–1.02)
Postoperative PI–LL	−0.0161	0.443	0.99 (0.95–1.03)
UIV level	−0.2167	0.790	0.80 (0.16–3.90)
PSO	−0.1363	0.786	0.88 (0.33–2.35)

Values in bold are considered statistically significant (*p* < 0.05). Abbreviations: BMI, body mass index; BMD, bone mineral density; HGS, hand grip strength; SS, sacral slope; PT, pelvic tilt; PI, pelvic incidence; LL, lumbar lordosis; PI–LL, pelvic incidence minus lumbar lordosis; UIV, upper instrumented vertebra; PSO, pedicle subtraction osteotomy; CI, confidence interval.

## Data Availability

Data to support the findings of this study are available upon reasonable request.
